# FUNGAL INFECTIONS, USE OF ANTIFUNGAL AGENTS, AND THE RISK OF ALZHEIMER’S DISEASE

**DOI:** 10.1093/geroni/igac059.002

**Published:** 2022-12-20

**Authors:** Arseniy Yashkin, Igor Akushevich, Anatoliy Yashin, Galina Gorbunova, Svetlana Ukraintseva

**Affiliations:** Duke University, Durham, North Carolina, United States; Duke University, Durham, North Carolina, United States; Duke University, Durham, North Carolina, United States; Duke University, Durham, North Carolina, United States; Duke University, Durham, North Carolina, United States

## Abstract

Alzheimer’s Disease (AD) is a complex neurodegenerative disorder leading to progressive cognitive decline and death. Accumulating evidence suggests that common adult infections, such as recurrent fungal infections, may play a major role in AD development. In this study we used administrative claims data, 1991-2017, from a 5% sample of U.S. Medicare beneficiaries age 65+ to study the potential relationship between fungal infections, use of antifungal drugs and AD onset. In unweighted analysis using the Cox model, we found that after accounting for demographic and health-related differences, the presence of fungal infections, independent of treatment increased the risk of AD [Hazard Ratio(HR):1.98; 95% Confidence Interval(CI):1.89-1.92]. The strength of the effect did not change when the population was restricted to Medicare beneficiaries with Medicare Part D (prescription drug) coverage in 2006+ (HR:1.99; CI:1.97-2.01). We then split the latter group into yearly split-episode data and calculated the medication possession ratios (MPR) of three major groups of antifungal agents: imidazole, triazole and polyenes and re-estimated our models. Although the effect associated with fungal infection did not change, higher MPRs of imidazole (HR:0.61; CI:0.48-0.76) and especially triazole (HR:0.37; CI:0.24-0.58) were found to be protective. In contrast, higher MPRs of polyenes were associated with increased risk (HR:1.32; CI:1.08-1.63). In summary, fungal infections were found to be strongly associated with AD risk with the effect being strongly modified by use of common antifungal treatments. More research is required to better define the biological mechanisms engendering these associations.

